# Possible effects of a free, healthy school meal on overall meal frequency among 10–12-year-olds in Norway: the School Meal Project

**DOI:** 10.1186/s13104-019-4418-6

**Published:** 2019-07-05

**Authors:** Frøydis N. Vik, Ida K. Næss, Kaia E. P. Heslien, Nina C. Øverby

**Affiliations:** Department of Public Health, Sport and Nutrition, University of Agder, Post-box 422, 4604 Kristiansand, Norway

**Keywords:** Children, Free school meal, Intervention, Meal frequency, Norway

## Abstract

**Objective:**

To evaluate possible effects of intake of a free, healthy school meal on overall meal frequency among 10–12-year-olds in Norway. This was evaluated using a quasi-experimental school-based intervention study assessing children’s meal frequency retrospectively using a questionnaire in two elementary schools in the southern part of Norway in 2014/15. Multiple logistic regression analyses with breakfast, lunch, dinner, supper as dependent variables were used.

**Results:**

A total of 164 children at baseline; 55 children in the intervention group and 109 children in the control group were included. The serving of a free school meal every day for 1 year did not improve the overall meal frequency in the intervention group compared to the control group. However, children in the intervention group had a lower odds of eating breakfast during weekends compared to the control group in crude analyses [odds ratio (OR) = 0.28 (95% confidence interval (CI) 0.09–0.84)] and in adjusted analyses [adjusted for baseline values, gender and socio-economic status) (OR = 0.15 (95% CI 0.03–0.72)]. Studies including larger study samples and therefore more statistical power are needed to further investigate possible effects of school meals on meal frequency.

*Trial registration* ISRCTN61703361, Date of registration: December 3rd, 2018, Retrospectively registered

## Introduction

There has been a rise in non-communicable diseases such as cardiovascular diseases, different types of cancers, and type 2 diabetes worldwide [1]. Diet is one of the key factors explaining this trend [1]. Great attention has therefore been given to children’s eating behaviour, as dietary habits acquired in childhood tend to persist into later ages [2, 3]. In general, a regular meal frequency is important for health [4]. Skipping meals is associated with an overall poorer diet quality [5], and a high meal frequency is inversely associated with childhood obesity [6–9].

Norwegian children consume at least one meal a day at school [10]. Moreover, all children attend school, and the vast majority (96%) attend public schools [11]. Therefore, public health prevention initiatives, such as nutrition interventions, organized in the school setting represent an ideal arena to promote healthy eating habits [12, 13]. In some Nordic countries a free, hot school meal is provided, and results from Finland show that eating a school lunch consisting of a main dish, salad and bread on a regular basis was associated with healthy eating [14].

There is currently no national scheme of school meals in Norwegian schools, and most school children bring a packed lunch from home in primary and secondary schools [15]. A cold meal consisting of bread with different kinds of spread is the most common food for school lunch. It is also part of the Norwegian food culture to eat a cold meal during lunchtime. To date, few studies have been conducted on the effects of a free school meal in Norway [16, 17]. Ask et al. found that serving a free lunch meal among 9th graders in Norway for 4 months did not improve the diet, i.e. intake of healthy food: fruit, vegetables, low-fat milk and wholegrain bread, or reduce the intake of snacks, sugar-sweetened beverages and candy/chocolate [17].

Good quality school meals have the potential to improve children’s overall diets and health [18]. The availability of healthy nutritious choices influences diet positively in school children [19]. A high-quality school lunch consumed by 11–16-year-old schoolchildren in Finland was found to be associated with a regular meal frequency and overall healthier eating patterns outside school [14]. This has to our knowledge not been assessed in Norway.

The aim of the present paper was to assess possible effects of the intake of a free, healthy school meal for 1 year on meal frequency among 10–12-year-olds in Norway.

## Main text

### Materials and methods

This study is a part of the School Meal Project [20]. A quasi-experimental non-randomized controlled school-based study was conducted in the southern part of Norway between 2014 and 2015, and an increased intake of healthy food at lunchtime among the children in the intervention group compared with the control group after 6 months has previously been reported [20]. The study was carried out among 5th, 6th and 7th grade school children, aged 10–12 years, from two different primary schools in a rural area. Data were collected at baseline (August/September 2014) with two follow-ups (January and June 2015). At all three data collections, the same questionnaire was completed in the classroom in the presence of a trained project worker (approximately 45 min). Meal frequency was assessed with items from a validated questionnaire [21].

#### Content of the intervention

A free, healthy school meal was served to all children in the intervention group every day from August 2014 until June 2015. The meal was prepared in accordance with current Norwegian dietary guidelines and consisted of wholegrain bread (at least 50% wholegrain) with a variety of healthy spread, and fruits and vegetables on the side [20]. The food was served on different trays, and the children helped themselves to the food they preferred. The food was consumed in the classroom, and the children ate together around one or two tables. Drinks were not served with the free meal, but children participating in the national school milk scheme drank milk. Children who did not drink milk were encouraged to drink water.

#### Sample and procedure

The School Meal Project was initiated by a local cook, who prepared and served the healthy school meal every day. The intervention school was therefore chosen based on convenience to make it feasible [20]. The school that served as a control school was chosen because it was equivalent to the intervention school in three ways: county, type of location (rural area) and school size.

**Fig. 1:**
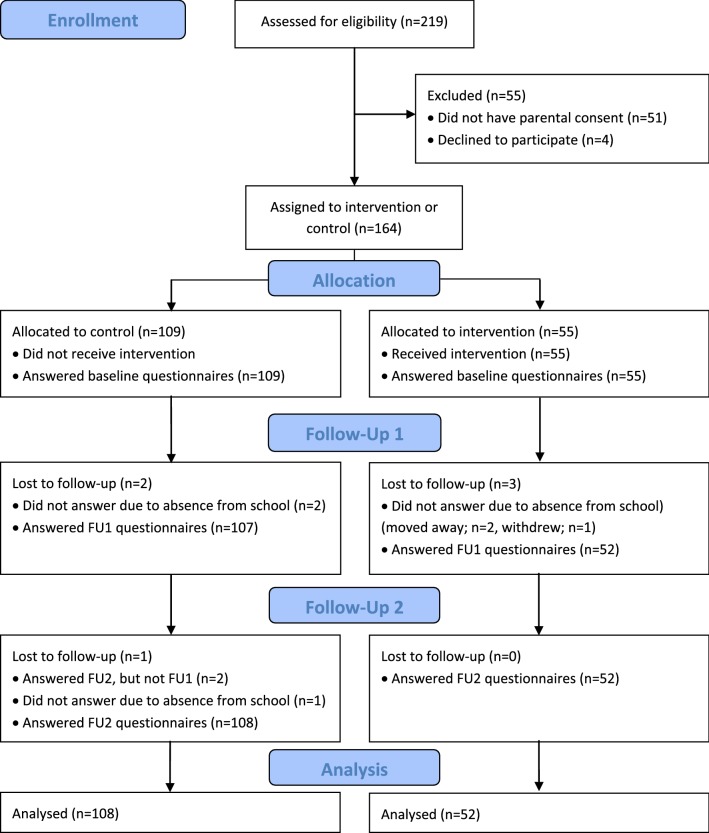
Flow diagram children

In total, 219 children from two different schools, aged between 10 and 12 years, and one of their parents were invited to participate. The headmaster at the intervention school was contacted first and after an agreement the control school was contacted. Information about the project was provided to parents at parent-teacher meetings and through written information. Written parental consent was given for 168 children. Four children did not want to participate, resulting in 164 participating children at baseline (response rate 75%), 55 in the intervention group (6th grade) and 109 in the control group (5th and 7th grade) from the same school as intervention group and 6th grade from the other school. A total of 154 parents participated (response rate 70%) at baseline. In the first follow-up, 159 children participated (response rate 73%). Of those lost to follow-up, three were from the intervention group and two from the control group. At the second follow-up, a total of 160 (response rate 73%) children responded. Those lost to follow-up were not present at school, had moved to another city or had withdrawn from the project (Fig. 1).

#### Measures

Age at the different time points was calculated from birth date, reported by the children. Parents’ level of education was assessed in the parent questionnaire by two items: “What is (a) your and (b) your spouse’s/partner’s highest level of completed education?” with four response options; “primary school (elementary school or lower secondary school)”, “upper secondary school”, “3–4 years of college or university” and “5 or more years of college or university” with an additional option for (b): “I do not have a spouse/partner”. The parents’ educational level was a proxy for socioeconomic status (SES). Both scores were combined, and dichotomized into “low SES” (both parents having completed primary school and upper secondary school) and “high SES” (at least one parent having completed 3–4 years and more than 5 years of college/university) [22].

Meal frequencies during weekdays was assessed by the following four questions: “How often do you eat breakfast/lunch/dinner/supper during the weekdays”? with the response options “Never”, “Once a week”, “Twice a week”, “Three times a week”, “Four times a week” and “Every day”. Meal frequencies during weekend days was assessed by the following four questions:” How often do you eat breakfast/lunch/dinner/supper during the weekend?” with response options “I don’t eat breakfast/lunch/dinner/supper during the weekend”, “Saturday *or* Sunday” and “Both Saturday and Sunday”. Each meal was assessed separately. Due to the small sample and little variation in the meal frequency (many children in the sample did eat the meals every day) we chose to dichotomize the meal variable. This is in line with several other studies [22, 23]. To assess the percentage of children eating main meals on weekdays, all four variables were dichotomized into either eating breakfast/lunch/dinner/supper every weekday (coded as 1) vs. not eating the respective meals every weekday (coded as 0). The variable eating main meals on weekend days, were dichotomized into either eating breakfast/lunch/dinner/supper both Saturday and Sunday (coded as 1) vs. not eating all the meals in the weekend days (coded as 0). All the weekdays variables were combined, and then dichotomized into three or four meals daily (coded as 1) vs. less than three meals daily (coded as 0). The same was done with the weekend days variables.

#### Statistics

All data were analysed using the SPSS statistical software package version 22.0. For all tests, P ≤ 0.05 was considered significant. Differences in age at baseline were analysed using Independent Samples T-Test. Differences in proportions of children eating different meals were analysed using Chi Square tests (Table 1). Multiple logistic regression analyses were conducted to calculate odds ratios with 95% confidence intervals of overall intervention effect on eating breakfast, lunch, dinner and supper and having a regular meal frequency on weekdays and during weekends separately. The independent “intervention/control”-variable was dummy coded with the intervention group coded as 1, and the control group coded as 0. Three regression models were performed on all outcome variables: the first model was unadjusted, while the second model was adjusted for baseline values, and the third model was adjusted for baseline values, gender, and parental education as a measure of SES as potential confounding factors.

**Table 1:** Descriptive statistics of the main meals (breakfast/lunch/dinner/supper) and regular meal frequency

	Baseline (N = 164)			Follow up 1 (N = 159)			Follow up 2 (N = 160)		
	Control	Intervention	P-value**	Control	Intervention	P-value**	Control	Intervention	P-value**
Total sample									
Breakfast every weekday %	91	85	0.298	93	92	0.974	91	89	0.653
Lunch every weekday %	93	91	0.670	94	98	0.288	91	90	0.942
Dinner every weekday %	88	93	0.346	90	94	0.413	86	89	0.680
Supper every weekday %	55	72	0.036*	58	58	0.962	52	65	0.106
Breakfast weekend days %	94	93	0.655	94	89	0.196	94	83	0.017*
Lunch weekend days %	65	68	0.666	61	54	0.395	54	44	0.262
Dinner weekend days %	93	91	0.695	93	92	0.813	85	94	0.113
Supper weekend days %	54	67	0.130	51	46	0.610	43	56	0.118
3 or 4 meals on weekdays %	82	79	0.627	86	82	0.602	85	81	0.478
3 or 4 meals on weekend days %	79	79	0.914	80	73	0.327	70	76	0.463

Meals eaten on weekend days are presented as eaten both Saturday and Sunday

* P ≤ 0.05

** Chi Square was used to test differences between the intervention group and the control group

### Results

At baseline, mean age in both groups were 11.1 years. There were 38% and 53% girls in the intervention and control group respectively. Further, 53% in the intervention group had high SES, while 63% had high SES in the control group.

There were no differences between the intervention group and the control group for the variables under study at baseline, except from eating supper every weekday. This difference was in favour of the intervention group (P = 0.036) (Table 1).

At the second follow-up the control group had a more frequent (favourable) breakfast consumption in the weekends compared to the intervention group (P = 0.017) (Table 1). Except from this finding, there were no differences regarding the proportions of children having the different meals every day between the intervention and control group at baseline, first and second follow up. The number of children having regular meals was generally high, ranging from 55 to 93%, with supper (evening meal) being the meal with the lowest numbers. The multiple logistic regression analysis showed that the intervention group had lower odds of eating breakfast in the weekends compared to the control group in the crude analyses [OR = 0.28 (95% CI 0.09–0.84)] (Table 2). After adjusting for baseline values, gender and SES, this association remained [OR = 0.15 (95% CI 0.03–0.72)] (Table 2).

**Table 2:** Odds ratios (OR) with 95% confidence intervals (CI) of overall intervention effect (intervention vs. control) on eating breakfast, lunch, dinner and supper in weekdays and weekend days, and having a regular meal frequency, in the total sample

	Model I (N = 160)			Model II (N = 160)			Model III (N = 160)		
	OR	CI	P-value	OR	CI	P-value	OR	CI	P-value
Breakfast every weekday	0.78	0.27–2.28	0.653	0.83	0.27–2.56	0.743	0.67	0.19–2.38	0.537
Lunch every weekday	0.96	0.31–2.97	0.942	0.94	0.30–2.92	0.916	1.06	0.33–3.39	0.926
Dinner every weekday	1.24	0.45–3.40	0.680	0.80	0.24–2.63	0.713	0.71	0.20–2.46	0.584
Supper every weekday	1.75	0.88–3.48	0.108	1.18	0.53–2.64	0.673	0.86	0.36–2.02	0.858
Breakfast weekend	0.28*	0.09–0.84	0.023	0.25*	0.08–0.79	0.019	0.15*	0.03–0.72	0.017
Lunch weekend	0.68	0.35–1.33	0.263	0.52	0.24–1.12	0.096	0.52	0.24–1.15	0.106
Dinner weekend	2.73	0.76–9.82	0.125	3.54	0.84–14.95	0.085	3.70	0.79–17.25	0.096
Supper weekend	1.70	0.87–3.31	0.119	1.40	0.61–3.24	0.426	1.23	0.50–2.96	0.653
3 or 4 meals weekdays	0.73	0.31–1.74	0.479	0.79	0.29–2.17	0.650	0.82	0.27–2.45	0.718
3 or 4 meals weekend days	1.33	0.62–2.88	0.464	1.36	0.58–3.19	0.481	1.17	0.49–2.83	0.725

*Model I* intervention vs. control; *Model II* intervention vs. control, adjusted for baseline values; *Model III* intervention vs. control, adjusted for baseline values, gender and SES

* P ≤ 0.05

### Discussion

The present study indicates that a free school meal for 1 year did not improve the overall meal frequency, and the results suggest that the intervention group skips breakfast during weekend to a larger degree than the control group.

We found no positive effect on meal frequency. This is not in line with the cross-sectional results from a German study among children, age 7–14 years, which found that a high participation rate in school lunch was positively associated with overall meal frequency [9]. Further, a Finnish study found that intake of school lunch was associated with more regular meal patterns, the availability of healthier foods at home and an overall healthier diet [14].

We found that children receiving the free school meal were less likely to eat breakfast during the weekend (Saturday and Sunday) compared with children in the control group. This was unexpected, and the result is difficult to interpret. To the best of our knowledge, no studies have previously examined the effect of a free school meal intervention in relation to meal frequency in the weekends. There is no rationale to why the intervention group should be more prone to skipping breakfast in the weekends. This may be caused by coincidence.

### Conclusions

Serving of a free healthy school meal every day for one year showed no improved effect on overall meal frequency. Studies including larger study samples and thereby higher statistical power are needed to further investigate possible effects of school meals on dietary habits outside school.

## Limitations

There are some limitations to this study. Firstly, most of the children in the present study reported to eat three or more meals during the day. The lack of variation in responses to the meal frequency questions is a major limitation of the study. Norwegian children have been known to have a regular meal frequency [24] and this study was conducted at two schools in a rural area, and children living in urban locations may skip meals more frequently than children in rural areas [25]. Secondly, the small sample size gives less statistical power. Thirdly, the non-randomized study design is a limitation. However, the second school was chosen because of similarities with the first school. Fourthly, the present study is based on self-reported data relying on memory which could introduce response bias. Also, all the children knew the aims of the intervention, and this may have influenced their responses. There are also strengths of the present study such as the one-year long duration of the intervention. The design with an intervention group and a control group, and the high participation rate are other strengths.

## Data Availability

The datasets used and/or analysed during the current study are available from the corresponding author on reasonable request.

## Abbreviations

SES: socioeconomic status
OR: odds ratio
CI: confidence interval
